# A Novel Neuropathological Subtype of Amyotrophic Lateral Sclerosis Characterised by Prominent Astroglial TDP‐43 Pathology

**DOI:** 10.1111/nan.70036

**Published:** 2025-09-10

**Authors:** Tomoyasu Matsubara, Yuishin Izumi, Yuki Hatanaka, Mika Takahara, Amuro Kondo, Kiyoshi Matsukura, Akira Arakawa, Toshihiro Haga, Ryosuke Miyamoto, Hiroya Naruse, Hiroyuki Morino, Tatsushi Toda, Shigeo Murayama, Yuko Saito

**Affiliations:** ^1^ Department of Neuropathology (The Brain Bank for Aging Research), Tokyo Metropolitan Institute for Geriatrics and Gerontology Tokyo Japan; ^2^ Department of Neurology Tokushima University Graduate School of Biomedical Sciences Tokushima Japan; ^3^ Department of Neurology Teikyo University School of Medicine Tokyo Japan; ^4^ Department of Diagnostic Pathology Teikyo University Hospital Tokyo Japan; ^5^ Department of Neurology, Graduate School of Medicine The University of Tokyo Tokyo Japan; ^6^ Department of Medical Genetics Tokushima University Graduate School of Biomedical Sciences Tokushima Japan; ^7^ Brain Bank for Neurodevelopmental, Neurological and Psychiatric Disorders United Graduate School of Child Development, Osaka University Osaka Japan

**Keywords:** amyotrophic lateral sclerosis, astrocyte, autopsy, flail arm syndrome, motor neuron disease, TDP‐43

## Abstract

- This study identified a novel amyotrophic lateral sclerosis subtype with prominent astroglial phosphorylated TDP‐43 inclusions and minimal neuronal inclusions.
- The patients shared a clinical phenotype of flail arm variant of ALS.
- These observations suggest a more critical role for astroglia than previously recognised.

**Abbreviations**
ALS: amyotrophic lateral sclerosis
BBAR: brain bank for aging research
FALS: familial ALS
GFAP: glial fibrillary acidic protein
MRI: magnetic resonance imaging
NCIs: neuronal cytoplasmic inclusions
PCR: polymerase chain reaction
pTDP‐43: phosphorylated TDP‐43
TDP‐43: transactivation response DNA‐binding protein 43 kDa

Amyotrophic lateral sclerosis (ALS) is a progressive neurodegenerative disease affecting both upper and lower motor neurones, characterised by intracytoplasmic inclusions of transactivation response DNA‐binding protein of 43 kDa (TDP‐43), typically found in neurones and oligodendroglia [1, 2, 3, 4]. ALS shows heterogeneous phenotypes; some patients may experience rapid disease progression, whereas others may have a longer disease course, or some patients may display symptoms confined to the motor system, whereas others may present with involvement of multiple systems [5, 6]. Nevertheless, the mechanisms underlying the differential involvement of neurones and glia, as well as the phenotypic variability observed among patients with ALS, remain poorly understood. Addressing this gap requires exploring the potential role of nonneuronal elements in the pathomechanism of the disease. Herein, we describe two cases of a distinct ALS subtype characterised by prominent astroglial TDP‐43 inclusions.

## Patient 1

A 72‐year‐old woman presented with proximal‐dominant bilateral upper limb weakness that began at the age of 70. The patient had no remarkable medical or family history of neurological diseases. One year after symptom onset, a neurological examination revealed marked muscle weakness and atrophy in the upper extremities, whereas the lower extremities showed only mild weakness of the distal muscles. The jaw and deep tendon reflexes were normal and decreased, respectively. The plantar responses were also normal. No cognitive dysfunction, cranial nerve abnormalities, extrapyramidal symptoms, ataxia, sensory deficits or autonomic dysfunctions were observed. Needle electromyography demonstrated active and chronic denervation with fasciculation potential in the brainstem, cervical, and lumbar regions. Spirometry revealed a reduced slow vital capacity of 47.9% of predicted. Brain and cervical magnetic resonance imaging (MRI) revealed no significant findings. The patient was diagnosed with flail arm variant of ALS. Later, the patient developed a dropped head due to neck muscle weakness, requiring a neck collar. The patient died of aspiration pneumonia at the age of 72. The disease duration was 2.3 years, and the patient remained ambulatory with minimal assistance throughout the clinical course.

## Patient 2

A 66‐year‐old man presented with muscle weakness that appeared in the right arm at the age of 55 and extended to both arms within a year. The patient had no remarkable medical or family history of neurological disease. One year after symptom onset, a neurological examination revealed muscle weakness and atrophy with fasciculations confined to the bilateral upper extremities. He had normal jaw reflexes, deep tendon reflexes in the extremities and plantar responses. Other examinations revealed no abnormalities. Needle electromyography demonstrated active and chronic denervation with fasciculation potential in the brainstem, cervical and lumbar regions. Spirometry showed normal respiratory function. Brain and cervical MRI revealed no significant findings. The patient was diagnosed with flail arm variant of ALS. By the age of 60, the patient developed dysarthria, dysphagia and respiratory failure, eventually becoming bedridden. Subsequently, at the age of 60, he underwent gastrostomy and tracheostomy with invasive ventilation. The patient died of pneumonia at the age of 66. The disease duration was 11 years (ventilator‐free survival time: 5 years), and the patient maintained the ability to communicate throughout the clinical course.

**FIGURE 1:**
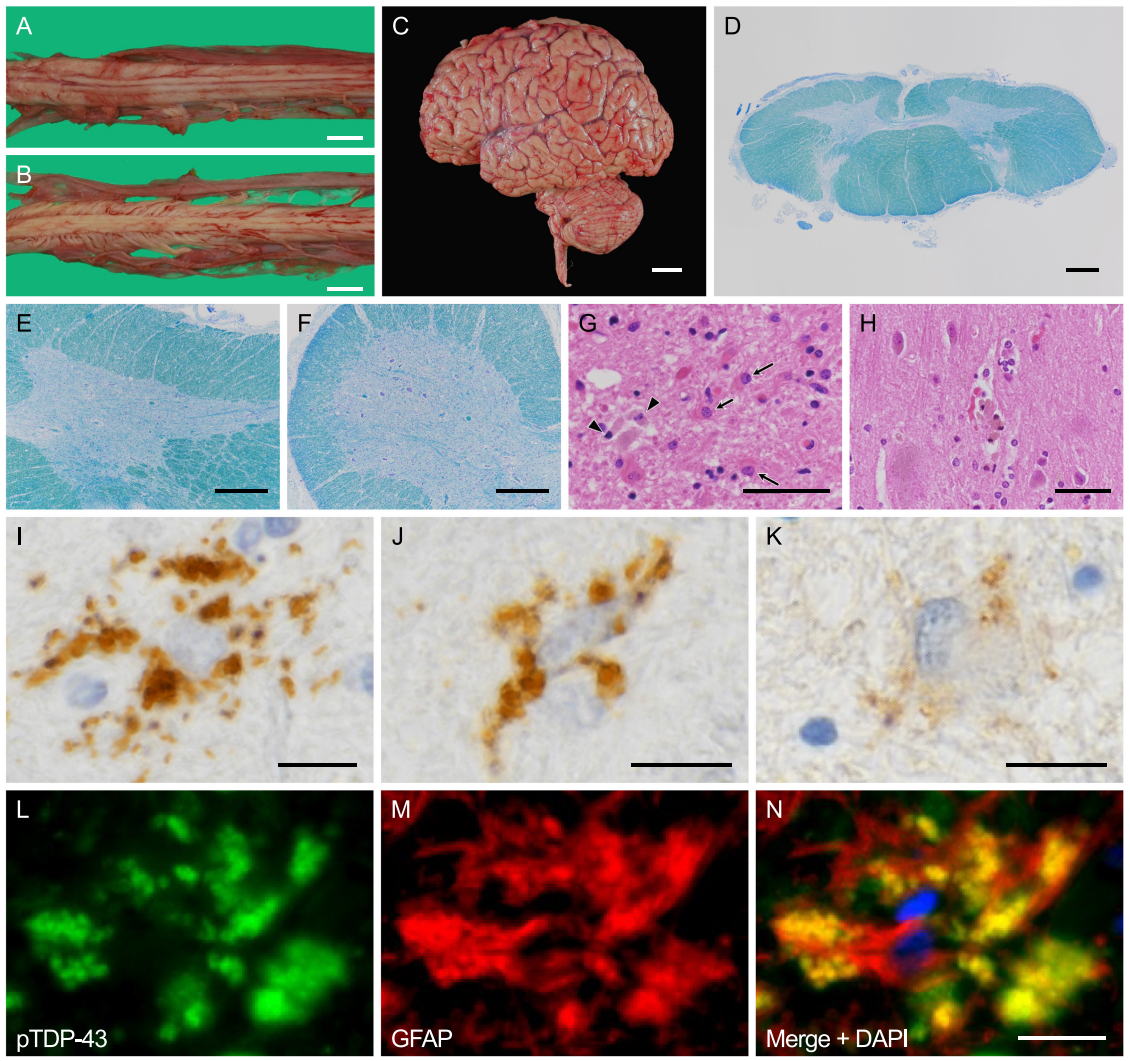
Pathological findings (Patient 1). Macroscopic examination reveals selective atrophy of the anterior cervical nerve roots (A) and relative sparing of the posterior cervical nerve roots (B). There is no noticeable atrophy in the cerebrum or the cerebellum (C). The cervical spinal cord shows no apparent decrease in myelin stainability in the corticospinal tract, with Klüver–Barrera staining (D). The cervical spinal cord anterior horn shows severe neuronal loss (E), while the lumbar anterior horn shows relatively mild neuronal loss (F) with Klüver–Barrera staining. Marked gliosis is observed, especially astroglia with eosinophilic processes (arrow), and the occasional accumulation of macrophages (arrowhead) is observed in the anterior horn of the spinal cord with haematoxylin and eosin (HE) staining (G). Occasional neuronophagia is observed in the precentral gyrus with HE staining (H). Characteristics of the astroglial inclusions: The pTDP‐43‐immunoreactive astroglial inclusion is observed and shows small globular aggregates densely populating the perikarya of astroglia and extending into the proximal parts of the astroglial processes in the spinal cord anterior horn (I: Patient 1; J: Patient 2). The astroglial inclusion partially shows immunoreactivity for ubiquitin in the spinal cord anterior horn (K: Patient 2). Double immunofluorescence staining reveals that the pTDP‐43‐immunoreactive structures are within the glial fibrillary acidic protein (GFAP)‐immunoreactive cell and partially colocalised with GFAP (L–N: Patient 1). Scale bars: (A, B) 1 cm; (C) 2 cm; (D) 1 mm; (E, F) 500 μm; (G, H) 50 μm; (I–N) 10 μm.

**FIGURE 2:**
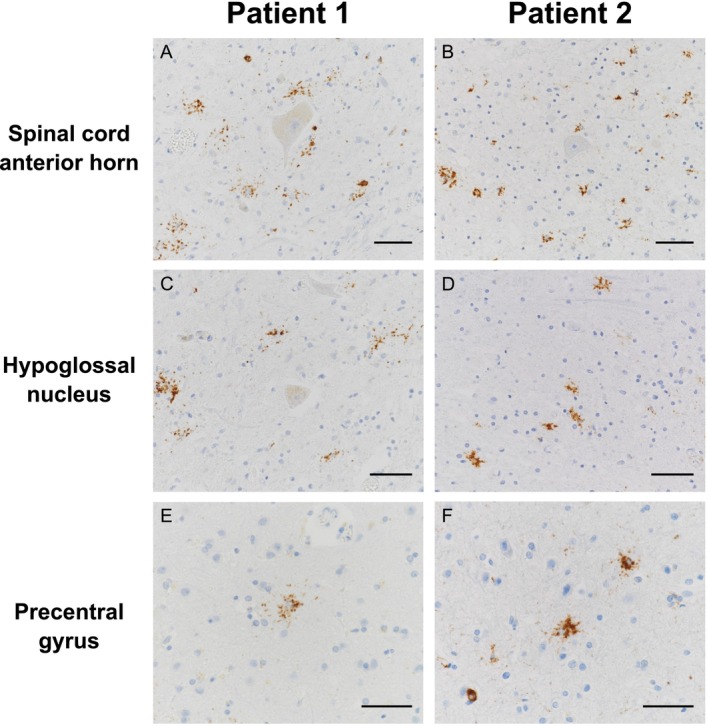
Distribution of the inclusions in each case. The pTDP‐43‐immunoreactive astroglial inclusions are abundant in the spinal cord anterior horns (A: Patient 1; B: Patient 2), and the hypoglossal nuclei (C: Patient 1; D: Patient 2), while preserved neurones have no pTDP‐43 immunoreactivity in these regions (A–D). The pTDP‐43‐immunoreactive astroglial inclusions are also observed in the precentral gyrus (E: Patient 1; F: Patient 2). Scale bars: (A–F) 50 μm.

The brain weights were 1074 and 1180 g for Patients 1 and 2, respectively. Details of the neuropathological examination methods are provided as Supporting Information. The patients had common histopathological features consistent with ALS. Macroscopically, the patients showed selective atrophy of the ventral spinal roots and the anterior horn (Figure 1A,B), whereas the cerebrum and the cerebellum did not appear to be affected (Figure 1C). Microscopically, the patients showed obvious lower motor neurone loss and gliosis, especially in the anterior horn of the spinal cord and hypoglossal nuclei, whereas degeneration of the corticospinal tract was obscure (Figure 1D–G). No Bunina bodies were observed. The brainstem nuclei innervating the extraocular muscles were well preserved. In the precentral gyrus, the patients exhibited scattered neuronophagia, suggesting a reduction in Betz cells (Figure 1H). Neuronal loss and gliosis were not evident in other regions, including the neocortical areas outside the precentral gyrus, limbic system, basal ganglia or cerebellum. The immunohistochemical analysis revealed the presence of pTDP‐43‐immunoreactive inclusions predominantly in the degenerated regions. Of note, the pTDP‐43‐immunoreactive inclusions were observed mainly in glial cells, especially in the astroglia (Figures 1I,J and 2). pTDP‐43 immunoreactivity revealed small globular aggregates densely populating the perikarya of astroglia and extending into their proximal processes. Some of these globular inclusions were also immunoreactive for ubiquitin (Figure 1K). The pTDP‐43‐immunoreactive structures were colocalised with glial fibrillary acidic protein (GFAP), confirming that they were astroglial inclusions (Figure 1L–N). No comparable globular inclusions were detected in oligodendroglia or microglia. The inclusions showed no immunoreactivity for phosphorylated tau or α‐synuclein. In addition, these inclusions were often observed around neurones (Figure 2A–C), but not around blood vessels or on the pial surface. In contrast, few classical oligodendroglial cytoplasmic inclusions and neuronal cytoplasmic inclusions were observed (Figure 2).

The detailed distribution of the pTDP‐43‐immunoreactive intracytoplasmic inclusions in the patients is summarised in Table 1. Astroglial cytoplasmic inclusions were especially abundant in the lower motor neurone regions and consistently predominated in other regions examined, except in the precentral gyrus of Patient 2. In the lower motor neurone regions, very few neuronal cytoplasmic inclusions were observed despite a certain degree of neuronal survival. A few neuronal intranuclear pTDP‐43‐immunoreactive inclusions were observed only in the putamen of Patient 2. Age‐related protein accumulation, including amyloid β and phosphorylated tau, was minimal (Patient 1: Thal Phase 3, CERAD score Moderate, Braak NFT Stage I; Patient 2: Thal Phase 0, CERAD score None, Braak NFT Stage I), and no α‐synuclein‐immunoreactive inclusions or argyrophilic grains were observed.

**TABLE 1:** Neuropathological profile of ALS with prominent astroglial TDP‐43 pathology.

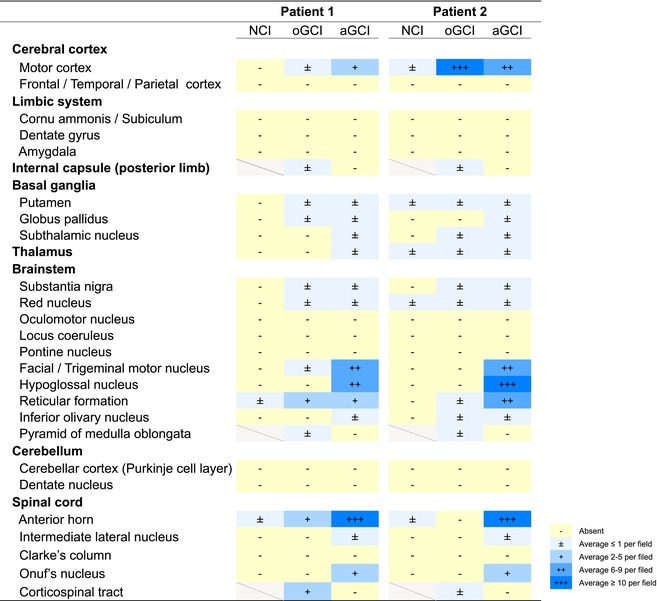

Abbreviations: aGCI, astroglial cytoplasmic inclusions; NCI, neuronal cytoplasmic inclusions; oGCI, oligodendroglial cytoplasmic inclusions.

Genomic DNA was extracted from autopsied brain specimens of two patients, and whole‐exome sequencing and repeat‐primed PCR for *C9orf72* hexanucleotide repeat expansions were performed as detailed in the Supporting Information. No pathogenic variants in the genes currently known to be associated with ALS were observed. No hexanucleotide repeat expansion was identified in *C9orf72*.

The present study highlights a distinct neuropathological subtype of ALS characterised by unique astroglial pTDP‐43 inclusions. ALS usually exhibits pTDP‐43 inclusions in neurones or oligodendroglia [3, 4]. In contrast, the patients exhibited abundant pTDP‐43 accumulation within astroglia, as demonstrated by immunohistochemistry, which exceeded the amount observed in neuronal and oligodendroglial inclusions. In addition, they shared the clinical phenotype of the flail arm variant of ALS.

The hallmark of these cases was an abundance of astroglial inclusions. Astroglial cytoplasmic inclusions have been previously described in familial ALS (FALS) with specific mutations in the *TARDBP* gene, which encodes the TDP‐43 protein. For example, astroglial inclusions have been observed in the anterior horn of the spinal cord of patients with FALS with a *TARDBP* p.N345K mutation [7] or in the motor cortex of patients with FALS with a *TARDBP* p.G357S mutation [8]. In contrast, our patients were sporadic and carried no *TARDBP* mutations. Thus, we have identified ALS with prominent astroglial inclusions as a subtype of sporadic ALS.

Despite pTDP‐43 accumulation within astroglia, the affected regions also exhibit neuronal loss. This phenomenon suggests that pTDP‐43 aggregates within astroglia may exert toxic effects on neurones, although potential alternative mechanisms that could challenge the idea of a primary astroglial pathogenic role warrant consideration. One could hypothesise that the astroglial inclusions stem from the propagation of TDP‐43 from neurones, given the networking between astroglia and neurones [9, 10]. Alternatively, the astroglial inclusions may result from astroglial uptake of pTDP‐43 released extracellularly during cell death or exocytosis, reflecting the inherent phagocytic abilities of astroglia [11, 12]. For these two hypotheses to be valid, the accumulation of pTDP‐43 in neurones should precede its accumulation in the astroglia. However, in the present cases, even in regions where neurones are relatively preserved, neuronal cytoplasmic inclusions are extremely rare. These observations suggest a potential role of astroglia in the pathomechanism of the disease and may raise the possibility of a noncell autonomous neuronal death mechanism [13].

Both patients had the flail arm variant of ALS, which is a rare form characterised by symptoms primarily affecting the upper limbs and accounts for approximately 5% of all ALS cases [14, 15]. The occurrence of this phenotype in both patients raises the possibility of a shared underlying mechanism. The cell‐type–specific localisation of TDP‐43 aggregates may also contribute to the clinical features. If so, recognising this neuropathological variation could be important when developing targeted therapies.

The limitation of this study is the small number of patients and the lack of a formal quantitative evaluation of neuronal loss. Nevertheless, the distinct clinicopathological features shared among these cases indicate the presence of a unique ALS subtype, warranting future research that combines further pooling of similar cases with rigorous quantitative analyses to elucidate the factors affecting the TDP‐43 accumulation patterns.

We describe a distinct neuropathological phenotype characterised by the presence of astroglial pTDP‐43 inclusions with minimal neuronal inclusions. Astroglia may potentially contribute to motor neurone degeneration and the clinical heterogeneity observed in ALS.

## Author Contributions

T.M. designed and conceptualised the study, analysed and interpreted the data and drafted the manuscript. S.M. and Y.S. designed and conceptualised the study, analysed and interpreted the data and revised the manuscript. Y.I., Y.H., M.T., A.K., K.M. and R.M. collected the clinical data, interpreted the data and revised the manuscript. A.A., T.H., H.N., H.M. and T.T. analysed and interpreted the data and revised the manuscript. All authors read and approved the final manuscript.

## Ethics Statement

This retrospective descriptive study was approved by the institutional review boards of the Tokyo Metropolitan Institute for Geriatrics and Gerontology. Informed consent was obtained from the families of the patients in accordance with the protocol of each institution. The study was conducted in accordance with the principles of the Declaration of Helsinki.

## Conflicts of Interest

The authors declare no conflicts of interest.

## Supporting information


**Data S1:** Supporting information.

## Data Availability

The datasets of this study are available from the corresponding author upon reasonable request.
