# Supplementary material for: A Novel Neuropathological Subtype of Amyotrophic Lateral Sclerosis Characterised by Prominent Astroglial TDP‐43 Pathology
Source: Neuropathol Appl Neurobiol. 2025 Sep 10;51(5):e70036. doi: 10.1111/nan.70036 (PMC12422077; doi:10.1111/nan.70036)
Supplement: Supplementary file 1 — Data S1: Supporting information. [file NAN-51-e70036-s001.docx]

**Materials and Methods**

**Patients**

Two patients with amyotrophic lateral sclerosis (ALS) and prominent transactivation response DNA-binding protein 43 kDa (TDP-43) astroglial inclusions were identified among 73 consecutive ALS cases (2.7%) from a series of brains and spinal cords registered to the Brain Bank for Aging Research (BBAR), Tokyo Metropolitan Institute for Geriatrics and Gerontology, Japan, between 2017 and 2021.

**Histopathology and immunohistochemistry**

Neuropathological examinations were performed according to the BBAR protocol as previously reported [1, 2]. In brief, 6 µm thick sections were cut from formalin-fixed, paraffin-embedded blocks obtained from representative anatomical regions in the brain and spinal cord; these sections were stained with haematoxylin and eosin (HE), Klüver–Barrera, and Gallyas–Braak staining. Subsequently, the immunoreaction product deposits on the immunohistochemically stained sections were visualised using a Ventana BenchMark GX autostainer (Ventana Medical Systems, Tucson, AZ, USA) and an I-View Universal DAB Detection Kit (Roche, Basel, Switzerland). The primary antibodies were used against phosphorylated TDP-43 (pTDP-43) pSer409/410 (clone 11-9; mouse-monoclonal, dilution 1:10,000; Cosmo Bio, Tokyo, Japan), phosphorylated tau (clone AT8; mouse-monoclonal, dilution 1:1,000; Innogenetics, Ghent, Belgium), amyloid β 11-28 (clone 12B2; mouse-monoclonal, dilution 1:50; IBL, Gunma, Japan), and phosphorylated α-synuclein (clone pSyn#64; mouse-monoclonal, dilution 1:10,000; FUJIFILM Wako Pure Chemical Corporation, Osaka, Japan).

For each tissue section region, the number of pTDP-43-immunoreactive cytoplasmic inclusions was counted under 10× objective magnification and graded as follows: -, absent; ±, average ≤ 1 per field; +, average 2–5 per field; ++, average 6–9 per field; and +++, average ≥ 10 per field.

Co-existing age-related pathologies were assessed according to established neuropathological criteria. Specifically, amyloid β (Aβ) plaque pathology was evaluated according to the Thal phase for Aβ deposition [3] and the neuritic plaque scoring protocol from the Consortium to Establish a Registry for Alzheimer's Disease (CERAD) [4]. The topographical distribution of neurofibrillary tangles was staged according to the Braak staging system [5, 6]. The distribution of Lewy body related α-synuclein pathology was evaluated based on the BBAR Lewy staging system [7, 8], and the distribution of argyrophilic grain pathology was assessed according to the Saito staging system [9].

**Double immunostaining**

Double immunofluorescence staining was performed using the antibodies against pTDP-43 (as described above), and antibodies against glial fibrillary acidic protein (GFAP) (rabbit-polyclonal, dilution 1:500; Abcam, Cambridge, UK). The primary antibodies were detected using Alexa Fluor^™^ 488-conjugated goat anti-mouse IgG and Alexa Fluor^™^ 594-conjugated goat anti-rabbit IgG (Thermo Fisher Scientific, Waltham, MA, USA). The samples were observed under a confocal laser scanning microscope (STELLARIS 5; Leica Microsystems, Wetzlar, Germany).

Double immunostaining was also performed for pTDP-43 and ionized calcium-binding adapter molecule 1 (Iba1) (rabbit-polyclonal, dilution 1:2,000; FUJIFILM Wako Pure Chemical Corp., Osaka, Japan) as well as for pTDP-43 and human olig2 (rabbit-polyclonal, dilution 1:50; IBL, Gunma, Japan).

**Genetics**

Genomic DNA was extracted from the autopsied brain specimens. Whole-exome sequence analysis of the two patients was performed using the SureSelect Human All Exon V7 kit (Agilent, Santa Clara, CA, USA) and the Twist Human Core Exome 2.0 kit (Twist Bioscience, South San Francisco, CA, USA). Sequencing was conducted on the NovaSeq 6000 and NovaSeq X (Illumina, San Diego, CA, USA) platforms at Macrogen Japan Corp (Tokyo, Japan). The obtained exome sequence data were analysed following a previously established workflow [10]. We examined variants in genes associated with ALS or frontotemporal lobar degeneration (FTLD), including *SOD1, FUS, TARDBP, SETX, VAPB, SPG11, OPTN, VCP, UBQLN2, ALS2, SIGMAR1, PFN1, ERBB4, HNRNPA1, HNRNPA2B1, MATR3, ANXA11, TBK1, KIF5A, TIA1, GLT8D1, SPTLC1, CCNF, CYLD, ANG, FIG4, CHMP2B, TUBA4A, NEK1, DAO, SPAST, GLE1, SS18L1, CHCHD10, SQSTM1, GRN, MAPT, DCTN1, ERLIN1, ERLIN2, HSPB1, CACNA1H, SORD, SYNE1, VRK1, PNPLA6, NEFH, EWSR1, PRPH*, and *TAF15*. Utilising population databases including the Genome Aggregation Database (gnomAD v3) and the Japanese Multi Omics Reference Panel (jMorp; 38KJPN), we focused on rare variants with a minor allele frequency (MAF) of < 0.01. All rare non-synonymous, nonsense, insertion/deletion, or splice-site variants were evaluated through whole-exome sequencing. Repeat expansion mutations in *C9orf72* were detected using repeat-primed polymerase chain reaction analysis.

**References**

1. A. Uchino, M. Takao, H. Hatsuta, et al., “Incidence and extent of TDP-43 accumulation in aging human brain,” *Acta Neuropathol Commun* 3, no. 1 (2015): 35,<https://doi.org/10.1186/s40478-015-0215-1>.

2. T. Matsubara, M. Kameyama, N. Tanaka, et al., “Autopsy Validation of the Diagnostic Accuracy of (123)I-Metaiodobenzylguanidine Myocardial Scintigraphy for Lewy Body Disease,” *Neurology* 98, no. 16 (2022): e1648-e1659,<https://doi.org/10.1212/WNL.0000000000200110>.

3. D. R. Thal, U. Rüb, M. Orantes and H. Braak, “Phases of A beta-deposition in the human brain and its relevance for the development of AD,” *Neurology* 58, no. 12 (2002): 1791-1800,<https://doi.org/10.1212/wnl.58.12.1791>.

4. S. S. Mirra, A. Heyman, D. McKeel, et al., “The Consortium to Establish a Registry for Alzheimer's Disease (CERAD). Part II. Standardization of the neuropathologic assessment of Alzheimer's disease,” *Neurology* 41, no. 4 (1991): 479-486,<https://doi.org/10.1212/wnl.41.4.479>.

5. H. Braak and E. Braak, “Neuropathological stageing of Alzheimer-related changes,” *Acta Neuropathologica* 82, no. 4 (1991): 239–259,<https://doi.org/10.1007/BF00308809>.

6. H. Braak, I. Alafuzoff, T. Arzberger, H. Kretzschmar and K. Del Tredici, “Staging of Alzheimer disease-associated neurofibrillary pathology using paraffin sections and immunocytochemistry,” *Acta Neuropathologica* 112, no. 4 (2006): 389-404,<https://doi.org/10.1007/s00401-006-0127-z>.

7. Y. Saito, A. Kawashima, N. N. Ruberu, et al., “Accumulation of phosphorylated alpha-synuclein in aging human brain,” *J Neuropathol Exp Neurol* 62, no. 6 (2003): 644–654,<https://doi.org/10.1093/jnen/62.6.644>.

8. Z. I. Tanei, Y. Saito, S. Ito, et al., “Lewy pathology of the esophagus correlates with the progression of Lewy body disease: a Japanese cohort study of autopsy cases,” *Acta Neuropathologica* 141, no. 1 (2021): 25-37,<https://doi.org/10.1007/s00401-020-02233-8>.

9. Y. Saito, N. N. Ruberu, M. Sawabe, et al., “Staging of argyrophilic grains: an age-associated tauopathy,” *J Neuropathol Exp Neurol* 63, no. 9 (2004): 911–918,<https://doi.org/10.1093/jnen/63.9.911>.

10. H. Naruse, H. Ishiura, J. Mitsui, et al., “Burden of rare variants in causative genes for amyotrophic lateral sclerosis (ALS) accelerates age at onset of ALS,” *J Neurol Neurosurg Psychiatry* 90, no. 5 (2019): 537-542,<https://doi.org/10.1136/jnnp-2018-318568>.
